# Correspondence on NanoVar’s performance outlined by Jiang T. et al. in “Long-read sequencing settings for efficient structural variation detection based on comprehensive evaluation”

**DOI:** 10.1186/s12859-023-05484-w

**Published:** 2023-09-20

**Authors:** Cheng Yong Tham, Touati Benoukraf

**Affiliations:** 1 Cancer Science Institute of Singapore, National University of Singapore, Singapore, 117599 Singapore; 2 Division of BioMedical Sciences, Faculty of Medicine, Memorial University of Newfoundland, St. John’s, NL A1B 3V6 Canada

**Keywords:** NanoVar, Structural variation, SV calling, Benchmark, Long-read sequencing

## Abstract

A recent paper by Jiang et al. in *BMC Bioinformatics* presented guidelines on long-read sequencing settings for structural variation (SV) calling, and benchmarked the performance of various SV calling tools, including NanoVar. In their simulation-based benchmarking, NanoVar was shown to perform poorly compared to other tools, mostly due to low SV recall rates. To investigate the causes for NanoVar's poor performance, we regenerated the simulation datasets (3× to 20×) as specified by Jiang et al. and performed benchmarking for NanoVar and Sniffles. Our results did not reflect the findings described by Jiang et al. In our analysis, NanoVar displayed more than three times the F1 scores and recall rates as reported in Jiang et al. across all sequencing coverages, indicating a previous underestimation of its performance. We also observed that NanoVar outperformed Sniffles in calling SVs with genotype concordance by more than 0.13 in F1 scores, which is contrary to the trend reported by Jiang et al. Besides, we identified multiple detrimental errors encountered during the analysis which were not addressed by Jiang et al. We hope that this commentary clarifies NanoVar's validity as a long-read SV caller and provides assurance to its users and the scientific community.

## Background

The benchmarking of structural variation (SV) calling tools provides end-users with vital information for comparing and selecting the optimal tool and settings for SV detection in their research. Hence, it is important to ensure that benchmark analyses are accurate and fair to faithfully reflect the benefits and drawbacks of each tool. In a recent paper by Jiang et al. [1], a benchmark analysis was performed on long-read SV callers using simulated datasets of varying sequencing coverages. NanoVar [2], one of the long-read SV callers, was shown to perform poorly in the benchmark across all sequencing coverages, mostly due to a low recall rate of SVs. We argue that NanoVar's poor performance in Jiang et al. contradicts other independent studies that showed rather adequate results. For instance, Wu et al. [3] had utilized NanoVar for SV calling in 405 Chinese individuals and showed that 72% of SVs called by NanoVar overlapped with 67% of SVs called by Sniffles on average, suggesting comparable sensitivity of both tools. In the benchmark analysis of Dierckxsens et al. [4], NanoVar’s F1 scores ranged from 0.841 to 0.853 for three 20× simulated datasets, which are within 0.05 of the F1 scores of other SV callers. Furthermore, a recent paper by Cleal and Baird [5] benchmarked NanoVar with F1 scores of 0.922 and 0.898 for deletion and insertion SVs, respectively, using real data from the Genome in a Bottle (GIAB) consortium. The disparity of NanoVar’s performance between Jiang et al. and other studies [2–5] is concerning and warrants more investigation, especially for the prior and present users of NanoVar. This correspondence aims to validate NanoVar’s performance and investigate the underlying causes of its apparent poor performance in Jiang et al. benchmark.

## Nanovar benchmark

**Fig. 1:**
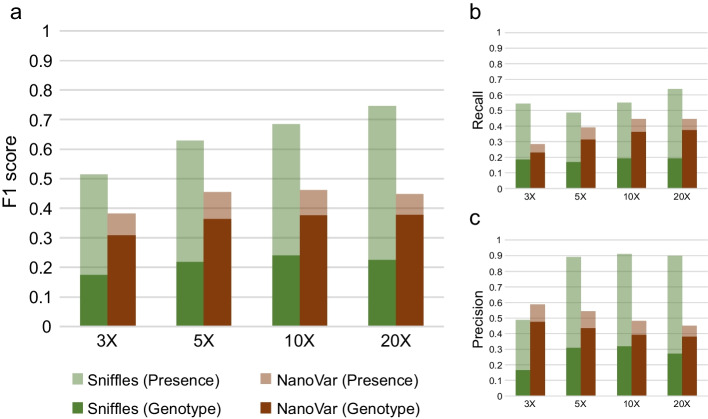
Benchmarking of NanoVar and Sniffles using simulated datasets. Bar plots showing the F1 score (**a**), recall (**b**) and precision (**c**) for SV calling by NanoVar and Sniffles for simulated long-read sequencing data with coverages of 3×, 5×, 10×, and 20×. The generation of SV simulated long-read sequencing datasets and tool benchmarking were carried out as stated by the protocol provided by Jiang et al. with several necessary modifications due to errors encountered during analysis (Please refer to *Availability of data and materials*)

To investigate the poor performance of NanoVar in Jiang et al., we regenerated the long-read simulation datasets and benchmarked NanoVar in accordance to the methods stated in Jiang et al. (https://github.com/SQLiu-youyou/The-commands-of-the-evaluation). For comparison, we have also included Sniffles [6] (described as one of the software with the highest performance) in the benchmarking. Due to errors raised during the benchmark analysis by Truvari [7] (v3.0.1), some SVs from each tool were filtered-out (Not mentioned in Jiang et al.), which will be discussed later. Despite employing the identical simulated datasets, our benchmarking results yielded better NanoVar performance scores than Jiang et al. (Fig. 1). As Jiang et al. did not provide the exact benchmark scores of NanoVar in their benchmark (Table S2 in Jiang et al.), we can only compare our results with the bar graphs in Fig. 2 of their publication. Jiang et al. showed that NanoVar acquired F1 scores of less than 0.1 for all sequencing coverages of 3×, 5×, 10×, and 20×, which is at least threefold lower than what we observed in our results, which had F1 scores of 0.38, 0.45, 0.46, and 0.45, respectively (Fig. 1a). The disparity of F1 score reporting is most likely explained by differences in SV recall, where we observed higher recall rates of 0.28, 0.39, 0.45, and 0.44 for respective sequencing coverages (Fig. 1b), as compared to less than 0.05 for all coverages in Jiang et al. Moreover, at 3× coverage, NanoVar achieved greater precision than Sniffles (Fig. 1c), which was not reflected in Jiang et al. NanoVar’s F1 scores of SV calling with genotype concordance was also observed to be higher in our results (Fig. 1a). Collectively, our repeated benchmark analysis using the same simulated datasets suggests that Jiang et al. may have underestimated NanoVar's performance.

We also observed different performance results for Sniffles. While our F1 scores of Sniffles for SV calling by presence are broadly in agreement with Jiang et al., our F1 scores for SV calling by genotype concordance were substantially lower (Fig. 1a). F1 scores for Sniffles (Genotype) ranged from 0.17 to 0.24 in our analysis, whereas Jiang et al. reported F1 scores ranging from 0.50 to 0.70, for sequencing coverages of 3× to 20×. Consequently, we observed that NanoVar (Genotype) has outperformed Sniffles (Genotype) by more than 0.13 in F1 scores across all sequencing coverages (Fig. 1a). Sniffles' reduced performance in SV genotyping was also consistent with the benchmarks of Dierckxsens et al. [4], where Sniffles’ genotype scores were at least 30% lower than other SV callers. These results suggest that Jiang et al. may have overestimated Sniffles' SV genotyping capability.

During our analysis, we made some changes to certain output files within the protocol described by Jiang et al. However, these changes were made in order to rectify the errors that we encountered. As these errors were not mentioned by Jiang et al., they were unanticipated while following their protocol, and we are uncertain how Jiang et al. had resolved them to allow successful completion of their analysis. The first error we encountered happened during the long-read simulation step using VISOR [8] (v1.1) where we obtained an empty BAM output file. After consulting with the author of VISOR, we discovered that the problem was with the “SHORtS.LASeR.bed” file provided by Jiang et al., in which the start coordinates of genomic regions in the file were “0”s instead of “1”s (c.f. https://github.com/davidebolo1993/VISOR/issues/18). The problem was resolved after we corrected the start coordinates of the file. The second error occurred when NanoVar was running on the simulated long-read BAM file produced by VISOR. The error happened because the read names of the simulated reads contained the comma (,) symbol, which resulted in a parsing error and prevented NanoVar from completing successfully. After removing the commas in the read names, NanoVar completed its run with no errors. As this was a necessary correction to obtain results from NanoVar, it is unclear how Jiang et al. had handled it and whether this influenced the results. The third error happened due to VCF file incompatibilities with Truvari for NanoVar and Sniffles. For NanoVar, an error was raised due to the presence of “>” or “.” symbols in the “SVLEN” field of some entries in the VCF file. These symbols were added by NanoVar to refine information on SV length, or nullify it for SVs with no lengths, respectively. When these symbols were omitted from the VCF file, Truvari ran successfully. For Sniffles, the error was caused by the “STRANDBIAS” string in the “FILTER” column of a few entries (< 50), and eliminating these entries resolved the problem. With the presentation of these VCF incompatibilities, it is plausible that there might be more nuances in the VCFs of NanoVar and Sniffles that impede an accurate assessment by Truvari. Taken together, we are uncertain how these fundamental errors were addressed by Jiang et al. and if they may have affected the results.

## Outlook

In conclusion, based on the Jiang et al. published materials, we were not able to entirely reproduce the results described by the authors’ benchmark. Indeed, our analysis performed on the same simulated datasets suggests an underestimation of NanoVar’s performance and an overestimation of Sniffles’ SV genotyping performance. We also encountered multiple errors while trying to replicate their analysis which might explain the discrepancy in results. We hope that the discussions provided here, as well as other studies [2–5], have clarified the performance of NanoVar as a long-read SV caller and provided the confidence for its continual use in research.

## Data Availability

The commands used to replicate the benchmark analysis by Jiang et al. (Including the changes mentioned) can be found at https://github.com/cytham/nv_benchmark_jiang. The Truvari results of each benchmark can be found at https://github.com/cytham/nv_benchmark_jiang/tree/main/truvari_summary.

## Abbreviation

SV: Structural variation
